# The role, readiness to change and training needs of the Athlete Health and Performance team members to safeguard athletes from interpersonal violence in sport: a mini review

**DOI:** 10.3389/fspor.2024.1406925

**Published:** 2024-05-06

**Authors:** Allyson Gillard, Margo Mountjoy, Tine Vertommen, Stephanie Radziszewski, Véronique Boudreault, Natalie Durand-Bush, Sylvie Parent

**Affiliations:** ^1^ Research Chair in Security and Integrity in Sport, Université Laval, Québec City, QC, Canada; ^2^ Department of Physical Education, Faculty of Education, Université Laval, Québec City, QC, Canada; ^3^ Interdisciplinary Research Center on Intimate Relationship Problems and Sexual Abuse (CRIPCAS), Montréal, QC, Canada; ^4^ Équipe Violence Sexuelle et Santé (ÉVISSA), Université du Québec à Montréal, Montréal, QC, Canada; ^5^ International Research Network on Violence and Integrity in Sport (IRNOVIS), Antwerp, Belgium; ^6^ Department of Family Medicine, McMaster University, Hamilton, ON, Canada; ^7^ Safeguarding Sport and Society, Center of Expertise Care and Well-Being, Thomas More University of Applied Sciences, Antwerp, Belgium; ^8^ Department of Physical Education, Ghent University, Ghent, Belgium; ^9^ Department of Kinanthropology, Faculty of Human Kinetics Sciences, Université de Sherbrooke, Sherbrooke, QC, Canada; ^10^ School of Human Kinetics, University of Ottawa, Ottawa, ON, Canada

**Keywords:** Athlete Health and Performance team members, interpersonal violence, sport, safe sport, safeguarding, duty of care, training

## Abstract

Safeguarding athletes from interpersonal violence (IV) in sport is an important topic of concern. Athlete Health and Performance (AHP) team members working with athletes have a professional, ethical, and moral duty to protect the health of athletes, prevent IV, and intervene when it occurs. However, little is known on their respective roles regarding IV in sport and their needs to fulfill their responsibility of safeguarding athletes. The aim of this review is to synthesize knowledge about the roles, readiness to change and training needs of AHP team members to navigate IV in sport. A total of 43 articles are included in the review. Results show that all AHP team members have a role to play in safeguarding athletes and should therefore be trained in the area of IV in sport. Overall, very little research has directly assessed AHP team members' needs to positively foster safety and eliminate IV in sport. There are common training needs for all types of AHP team members such as the ability to recognize signs and symptoms of IV in sport. However, there are also specific needs based on the role of the AHP team members such as ways of facilitating behavioural change for sport managers. Findings from this review are mostly experts' recommendations and should therefore be interpreted as such. The results of the review can guide the development of future research and recommendations.

## 1 Introduction

Recently, safeguarding all athletes from interpersonal violence (IV) in sport has become an important topic of concern (1, 2). IV in sport implies acts of psychological, physical, and sexual violence as well as neglect that occur between individuals within the sport community (3–5). Every individual, no matter their role in sport, can witness or commit IV toward athletes such as coaches and Athlete Health and Performance (AHP) team members (1, 6–8). Even though AHP team members can perpetrate IV themselves (9, 10), authors have recently suggested that AHP team members play an important role in the prevention and intervention regarding IV in sport (11–13). Indeed, while AHP team members do not necessary have a neutral position toward athletes, they typically have less control and authority over athletes than coaches (14–16). Their role is often related to taking care of the health and wellbeing of athletes and depending on their expertise, they may have professional and ethical responsibilities to protect athletes based on their regulatory body or college's policies and code of ethics. However, little is known on their respective roles and needs to fulfill their responsibility to safeguard athletes from IV in sport. The aim of this review is to synthesize knowledge about the roles, readiness to change and training needs of AHP team members to navigate IV in sport. AHP team members are classified in four types: (1) medical and paramedical staff, (2) sport managers, (3) mental performance (MP) and mental health (MH) practitioners and (4) strength and conditioning coaches and other training support staff.

## 2 Materials and method

Eligible articles were searched on EBSCO (SPORTDiscus), ProQuest, PsycINFO and PubMed databases in May 2023 using keywords specific to the research question (see Table 1). Articles that were (a) peer reviewed (2), published from January 2010 to May 2023 and (c) written in French or English were included in the review. A total of 2,355 articles were identified through the bibliographic search. Duplicates (*N* = 148) were removed, and 1,891 articles were judged irrelevant through screening title and abstract. After full text review of the remaining articles, 30 articles were included in the review. Other relevant sources (*N* = 13) known by the research team were considered important to the study aim and were also included in the review, for a total of 43 sources^1^.

**Table 1:** Keywords for bibliographic search.

Concepts (AND)	Sport	Athlete	Maltreatment	Stakeholders	Training
Keywords (OR)	Sport*	Athlet* Player*	Maltreat* Violen* Abus* Harass* Negl* Harm* Aggression Hazing Assault Victim* Bullying Prejudice	"Sport doctor” “Sport physicians” Physiotherapist* "physical therapist” Manager* "sport psychologist” "mental performance consultant” "physical trainers” "massage therapist” Nutritionist* Stakeholder* decision-maker” administrat*	Prevention Training Safeguard* Polic* Rules* Guideline* Education Program Intervention Protect Promotion awareness

*(asterisk) is the truncation symbol used in the bibliographic search.

## 3 Results and discussion

### 3.1 General findings

The need to train all AHP team members to safeguard athletes from IV in sport is raised by several authors (12, 17–21). Indeed, safeguarding athletes is perceived as a shared responsibility between all AHP team members (16, 21–25). Thus, preventing IV in sport depends on the commitment and collaboration of the collective AHP team members (12, 24). Each AHP team member must understands their roles, responsibilities, and obligations in this regard (23, 26). Consideration must also be given to their respective perspectives and needs in advancing safe sport (27). Evidence-based training for all AHP team members would allow a shared understanding of safe sport and coordinated actions (23). In this regard, some authors suggested topics that should be universally addressed in training regarding IV in sport (see Table 2). The next sections will detail the specific training needs for each type of AHP team members.

**Table 2:** Topics that should be universally addressed in training regarding IV in sport.

Topics
Definitions and prevalence of the different types of IV in sport (28, 29)
Signs and symptoms of IV in sport (30–32)
Risk factors and consequences of IV in sport (29, 33)
Perpetrators of IV in sport (29)
Challenges related to disclosure (29)
Appropriate response to a disclosure (31)
Reporting procedures and laws (29, 31)
Limits of interpersonal relationships between AHP team members and athletes (29, 31)
The modus operandi of the perpetrators of IV in sport (17)
Issues of diversity, equity and inclusion (30)

### 3.2 Medical and paramedical staff

Medical and paramedical staff includes actors responsible of athletes' health such as sport doctors and physiotherapists, to give a few examples. Seven articles addressed the role of medical and paramedical staff in safeguarding athletes from IV in sport, but none have directly assessed their needs to fulfill this role.

#### 3.2.1 Role

Medical and paramedical staff play an important role in athlete safeguarding, notably because of their professional duty of care to protect the physical and mental well-being of their patients, the development of confidential trusting therapeutic relationship with athletes and their frequent contact with them (33–35). Thus, involving medical and paramedical staff in athlete safeguarding would increase surveillance of the sporting environment (28). Based on their professional competencies, medical and paramedical staff also have the role to raise awareness and educate the sport community on the health impact of IV in sport (35). They should be leaders in the implementation of change through their role as health advocates for the development of safeguarding initiatives in sport organizations (35). Medical and paramedical staff should also support research in the field of safe sport in their sport organizations (35). Moreover, the therapeutic relationship they have with athletes gives them a privileged place to support athlete experiencing IV in their recovery (34). No article addressed the readiness to change of medical and paramedical staff.

#### 3.2.2 Training needs

The training needs of medical and paramedical staff regarding IV in sport fall into three main categories: (a) general knowledge about IV in sport, (b) prevention, and (c) intervention. Regarding general knowledge, medical and paramedical staff should be able to recognize IV in sport (12, 22), notably the general and sport-specific clinical presentation (signs and symptoms) of IV in sport (12, 18, 21, 24, 33–35). Medical and paramedical staff should also be able to recognize sport-related risk factors of IV in sport (21, 33) and the signs of an abusive or at-risk relationship (e.g., signs of grooming) (33).

Concerning prevention, medical and paramedical staff need the knowledge and abilities to prevent IV in sport (21, 24). They should also know how to screen for IV in sport by creating a climate of open and reassuring communication that encourages disclosure (22) and by developing screening tools specific to IV in sport (24). Given that medical staff have been involved as perpetrators in cases if IV in sport, it is also essential to educate them on existing safeguarding policies and best practices when performing their professional duties with athletes (e.g., requiring the presence of a chaperone during intimate clinical examinations with a minor athlete) to prevent IV in their interactions with athletes (30).

In terms of intervention, medical and paramedical staff should be trained to respond appropriately to an athlete's disclosure of IV in sport (12, 18, 21, 24, 30, 33, 35). They should also know what to do in case of a suspected or known case of violence toward an athlete (18, 21). Indeed, medical and paramedical staff need to know how to report allegations of IV in sport, the appropriate authorities to whom to report, and be aware of their reporting obligations and legal confidentiality issues (12, 24, 34, 35). It is also important to train them with the best practices for supporting, caring for and treating athletes affected by IV in sport (24, 33–35). Medical and paramedical staff must therefore develop clinical competencies in trauma-informed practice to prevent re-traumatization in their interventions with athletes who experience IV in sport (34, 35).

### 3.3 Sport managers

Sport managers are people in charge of organizational aspects of sport, for example administrators working for national organizations, university athletic program directors, and regional sport club managers. Among all types of AHP team members, sport managers^2^, are the ones who received the most attention in the literature regarding athlete safeguarding. A total of 21 articles addressed this group, four or which directly assessed sport managers' role in the prevention of IV in sport.

#### 3.3.1 Role

Results suggest that sport managers play a key role in promoting safe sport. Sport managers appear to have the primary responsibility for safeguarding athletes (36). Their organizational leadership is recognized as an essential element having the greatest impact on safe cultures and environments within sport (19, 37–39). Several authors also suggest that sport managers have a role in promoting and providing accessible evidence-based education programs on IV in sport to individuals involved in sport (e.g., AHP team members, coaches, athletes) (17, 18, 27, 29, 31, 40).

#### 3.3.2 Readiness to change

Organizational tolerance regarding IV in sport and conformity to traditional values in sport (e.g., masculinity, sport ethic, expertise) create conducive environments for all forms of IV in sport (15). For example, norms of masculinity and heteronormativity are associated with IV in sport and non-reporting of IV (14, 37, 41). According to high performance athletes, sport managers must prioritize athletes' holistic development and a safeguarding culture over performance (20). Such approach would place athletes' wellbeing first rather than short-term outcomes such as medals. This safeguarding culture must be an integral part of the organization's and not perceived as an add-on element imposed by external actors (e.g., funding instances) (19, 38). To remain relevant and effective, a safeguarding system must be continually reviewed and adapted, meaning that sport managers must commit to its sustainability (38). Managers should be ready to invest the necessary resources (e.g., material, financial, human) for the implementation and maintenance of a safeguarding system (38). Organizational change, commitment and distancing from traditional values are therefore necessary to promote safe sport (15).

#### 3.3.3 Training needs

When directly questioned about their training needs, sport managers stated that education is a key element to creating a safe sport environment (42). However, they think that training should go beyond awareness raising and explicitly address ways of facilitating behavioural change (42). Participants suggested various topics they would like addressed in training initiatives, such as policies, prevention, power dynamics, and reporting (how and when to report) (42). Sport managers also need to be better equipped to recognize signs and symptoms of IV in sport (32). Although training is perceived as one of the most important elements for the prevention of IV in sport, it is also one of the greatest challenges in terms of organizational capacity (43, 44). In addition, sport managers recommended having an independent body to monitor, investigate and manage complaints. While this may reflect their lack of resources and ability to address IV in sport by themselves (42), it also acknowledges the need for third-party independent entities to manage IV in sport.

The literature review points out on some actions that sport managers should take to foster safe sport, from which we can identify indirect training needs. First, it seems of prime importance that sport managers implement clear policies and codes of conduct (13, 22, 27, 28, 40). Codes of conduct should clearly define the limits of interpersonal relationships between all actors in sport, expected behaviours, and good practices, as well as unsafe practices and unacceptable behaviours (15, 18, 31, 33, 42, 45). Thus, sport managers should receive training on these subjects (e.g., concrete manifestations of IV in sport) to be able to develop clear and effective policies and codes of conduct. Moreover, policies and codes of conduct should target risk and protective factors of IV in sport related to both victimization and perpetration (13, 31), meaning that sport managers should also be able to identify these factors through training. It is important to mention that to be effective, policies and codes of conduct must be accompanied by enforcement strategies, such as clear disciplinary measures (42). Indeed, policies and codes of conduct are not sufficient themselves; it is also necessary to clearly define and enforced sanctions for each breach of ethics (15, 18, 22, 27, 36).

Second, sport managers should implement reporting mechanisms regarding IV in sport (15, 27, 29, 36, 46). Indeed, it is important that all sport actors know why, when, how and where to signal known or suspected cases of IV in sport (39, 42). Sport organizations must clearly establish procedures for reporting, managing allegations and responding appropriately to a report of IV (13, 18, 28, 30). Moreover, in order for a reporting mechanism to be effective, sport managers should be aware of the barriers to disclosure and take action to reduce them (34, 39).

Third, sport managers should offer support and appropriate resources to complainants and witnesses of IV in sport (13, 18, 22, 39). To ensure appropriate support, sport managers must recognize that each experience of IV in sport is unique and that complainants' needs will vary from one person to another. They should also work in collaboration with athletes experiencing IV in sport (34). Indeed, survivors of IV in sport are experts by experience and should therefore be involved in the development of safeguarding initiatives (34). However, to safely support and collaborate with survivors of IV in sport, it is essential that all those who interact with survivors are trained in trauma-informed approaches (e.g., understand stages of recovery, avoid stigmatizing language and re-traumatization, recognize signs of mental health challenges) (34).

Additionally, results of this literature review suggest that sport managers should understand the realities and needs of the different groups of athletes. Preventive measures to safeguard athletes are not “one-size-fits-all” and must take into consideration diversity and intersectionality (20, 26, 47). For instance, equity and inclusion should be part of safeguarding strategies (13, 20, 47). Importantly, Canadian high performance athletes belonging to an equity-deserving group consider that current safe sport measures are not appropriate for all athletes (47). They consider that the measures benefit normative athletes, as they were created for them, without taking into account the specific needs of various athlete groups (47). In this regard, athletes suggest training AHP team members to understand the needs and realities of different equity-deserving groups in sport (47).

### 3.4 Mental performance and mental health practitioners

MP and MH practitioners are professionals working with athletes to support their mental health, well-being, and performance. This group includes, for example, certified mental performance consultants, clinical psychologists and social workers. A total of seven articles addressed the role of MP and MH counsellors regarding IV in sport. Only two studies directly addressed this group's perceived needs in this area.

#### 3.4.1 Role

Several authors emphasized that MP and MH practitioners are in an unique position to safeguard athletes from IV in sport (11, 48, 49). Indeed, by the nature of their job, their knowledge of ethical practice as well as the neutral, confidential and trustworthy relationships they develop with athletes, they are well placed to recognize and intervene in cases of IV in sport (11, 16, 50, 51). Athletes are also more likely to disclose (directly or indirectly) their experience of IV in sport to practitioners they trust (11, 51). These practitioners, and especially mental performance consultants, often directly work with athletes on the sporting field, which gives them a privileged position to detect IV (11). They also have the professional expertise to implement prevention strategies (e.g., consult with coaches to help them shift controlling behaviours to autonomy-supportive ones) and minimize the impact of IV on athletes (e.g., train mental skills to cope with stress) (50). Moreover, because these practitioners are members of professional orders or associations, they have a duty of care to protect the physical and mental health of athletes (11, 16).

#### 3.4.2 Readiness to change

MP and MH practitioners in sport might be socialized in a performance-oriented culture which may lead them to not recognize or to accept certain abusive practices (e.g., constant yelling) (11). They may be former athletes themselves who have normalized certain practices because of a win-at-all-cost mentality, or they may be influenced by the very nature of their job to maximize sport performance (e.g., they may emphasize stress management, resilience, and mental toughness despite the toxicity of an environment) (11). It is therefore important that these practitioners remain aware of their own beliefs and biases, and engage in ongoing reflective practice to ensure their interventions promote and protect athletes' welfare above and beyond performance (11, 48, 52). While this has been raised specifically for MP and MH practitioners in the reviewed articles, we acknowledge that it can also apply for all types of AHP team members (i.e., medical and paramedical staff, sport managers, as well as physical trainers and other training support staff).

#### 3.4.3 Training needs

The studies that directly assessed MP and MH practitioners' knowledge regarding athlete safeguarding shows that these individuals are direct or indirect bystanders of IV in sport (51, 52). As a result, these practitioners wish to have better knowledge regarding safe sport, notably protection policies, regulations, or laws for minors or children (51). They expressed a need for more clarity regarding definitions of IV in sport and specific examples (51, 52). Results also show that practitioners want to be better equipped to recognize signs and symptoms of IV in sport and to identify appropriate intervention strategies (51, 52). Participants suggested that additional training in this area be integrated in academic programs and accreditation processes (51).

The need to include safe sport training in MP and MH practitioners' education programs has also been highlighted by several authors (11, 14, 53). They suggested that practitioners should be able to recognize general and sport-specific signs and symptoms of IV in sport (11, 16, 50). They must also recognize risk factors and high risk situations of IV in sport (11), and be equipped to implement prevention strategies (11, 16, 48) by understanding the root causes of IV in sport (11). Finally, MP and MH practitioners must know how to intervene in cases of IV in sport (11), while respecting existing laws and regulations. To ensure safe and effective interventions, they must also be trained in trauma-informed practices (16).

### 3.5 Physical trainers and other training support staff

Within the literature review, no articles addressing the role of strength and conditioning coaches and other training support staff regarding IV in sport were identified. This is surprising considering that over 10 years ago, Stirling and Kerr (51) highlighted the relevance of assessing the exposure and knowledge of IV in sport of various AHP team members, including strength and conditioning coaches and nutritionists. For instance, nutritionists could play an important role in detecting IV in sport since experiences of IV in sport have been correlated with disordered eating and eating disorders (54, 55). Body shaming has also been identified as a form of IV in sport and is used by some coaches for instrumental purposes to pressure athletes to reach the ideal weight for their sport (56, 57). As a result, nutritionists working with athletes exhibiting disordered eating should be equipped to recognize the signs of IV in sport, the mechanisms for reporting suspected or known cases of IV in sport, and to respond appropriately to an athlete's disclosure.

## 4 Conclusion

In conclusion, the necessity to provide evidence-based training to all AHP team members on the topic of IV in sport is widely supported by the scientific literature. However, to date, very little research has directly addressed the specific needs of each group of AHP team members in relation to IV in sport. As a result, the findings of this literature review are mostly experts' recommendations and may not necessarily reflect the real needs of AHP team members. Moreover, none of the articles reviewed gave specific insight on AHP team members' needs and expectations regarding the structure (e.g., duration, format, and frequency) of training on the area of IV in sport. More studies are required to document the specific needs of each type of AHP team members. This would allow the development of targeted training programs that respond to both general and unique or local needs. The results of the review can guide the development of future research and recommendations. Finally, recognizing AHP team members' role in the prevention of IV in sport, one should not assume that they can't participate to IV in sport or be the perpetrators of such harms themselves. Indeed, we acknowledge that AHP team members are part of a wider sport system often embedded in a win at-all-cost mentality that normalize IV in sport and in which there are inherent power relations between actors (11, 39, 42). This wider system can therefore have an impact on their propension and abilities to prevent and respond to IV in sport. Training AHP team members regarding IV in sport is therefore proposed as a way of promoting safe sport but will not be sufficient in itself. Prevention and intervention strategies should be implement in all levels of the sport socioecological model to establish an overall safeguarding culture (13, 58).

## Footnotes

^1^The included articles are marked by an asterisk (*) in the reference list.

^2^Articles addressing the role of sport organizations regarding IV in sport are included in the sport managers category since sport organizations are led by sport managers.
